# Self‐Assessment Organisational Readiness Tool (SORT) for Nursing Research Capacity Development: Results of a UK Delphi Study

**DOI:** 10.1111/inr.70084

**Published:** 2025-09-01

**Authors:** Parveen Ali, Julie Mcgarry, Ashfaque Ahmed Talpur, Joanne Cooper, Joanne Cooke

**Affiliations:** ^1^ School of Allied Health Professions, Nursing and Midwifery, University of Sheffield Sheffield South Yorkshire UK; ^2^ Doncaster and Bassetlaw Teaching Hospitals Doncaster UK; ^3^ School of Health Sciences, University of Nottingham Nottingham UK; ^4^ School of Allied Health Professions, Nursing and Midwifery, University of Sheffield Sheffield UK

**Keywords:** nurses, organisational support, research capacity building, research capacity development

## Abstract

**Aim:**

This Delphi study aimed to develop the Self‐assessment Organisational Readiness Tool (SORT), a prototype designed to assess healthcare organisations’ capacity to support nursing engagement in research activities.

**Background:**

Nurses are pivotal to evidence‐based healthcare, yet organisational barriers frequently hinder their engagement in research. Existing frameworks often lack the precision needed to evaluate organisational readiness to support nurses in research capacity building, leadership development and digital innovation. This study aims to bridge this gap through the development of the SORT framework.

**Methods:**

A structured Delphi consensus‐building process was conducted over three iterative rounds and supplemented by four expert working group workshops. Participants, identified through purposive sampling, included 43 professionals with expertise in research and development, evidence‐based practice and clinical and research leadership. Quantitative data were analysed using consensus thresholds (≥70% agreement), while qualitative feedback informed the iterative refinement of the tool.

**Findings:**

Consensus was achieved on 67 statements across five thematic areas: People‐centred research, releasing potential, research systems, careers and digitally enabled research. Key items highlighted the importance of skills development, organisational leadership and collaboration with academia and patient/public involvement. Limited consensus in areas such as monitoring progress and digital research highlights the need for further refinement. SORT demonstrated strong face validity, with plans for reliability testing and factor analysis in future work.

**Implications for Nursing Practice and Policy:**

SORT is designed to be used by nursing and R&D leaders, healthcare executives, managers and policymakers to evaluate and improve organisational readiness to support research aiming nurses in healthcare settings. It can inform strategic planning, workforce development and performance benchmarking within healthcare institutions. It will also serve as a guide to individual nurse respondents by increasing their awareness of various initiatives in their organisations that they should know about. SORT provides a practical framework for embedding research within healthcare organisations to support nursing practice. It offers organisations a tool to identify strengths and gaps in research capacity, informing workforce development and infrastructure planning. Policymakers can use SORT to benchmark readiness and align strategies with national objectives. While developed in the UK, SORT has the potential to support global efforts to advance nursing research capacity, as this is the first tool developed specially to measure organisational readiness to support nurses for research and has already generated interest in other countries and professions.

**Conclusion:**

SORT represents a significant step towards fostering a research culture within healthcare organisations. By aligning with the Chief Nursing Officer strategy in the UK context, it supports evidence‐based practice and enhances nursing's contribution to healthcare quality and innovation. Future work will focus on validating SORT's reliability and expanding its applicability across diverse healthcare settings.

## 1 Introduction

Nurses are the backbone of healthcare systems, providing up to 80% of direct patient care globally (World Health Organization 2020; Baig et al. 2022). Their role extends beyond clinical practice, encompassing care coordination, patient advocacy and research engagement to enhance patient safety, healthcare outcomes and professional development (Boaz et al. 2015; Ozdemir et al. 2015). Evidence suggests that research‐active organisations benefit from lower risk‐adjusted mortality rates, improved patient experiences and enhanced staff recruitment and retention (Jonker et al. 2020; Rees and Bracewell 2019; Ozdemir et al. 2015). Engaging nurses in research promotes evidence‐based practice, strengthens clinical decision‐making and supports the development of innovative healthcare interventions (Janerka et al. 2024). It also contributes to their professional growth, offering pathways for career advancement and leadership (Hare and Whitehouse 2022). Moreover, research plays a pivotal role in shaping health policies, providing robust evidence to guide resource allocation and healthcare planning (Anders 2021; Kirk Koyama 2018). This evidence can be used to influence policymakers and stakeholders to prioritise nursing issues and allocate resources accordingly.

Despite its significance, nurses, particularly those on the frontline, face multiple barriers to research participation (Jabonete and Roxas 2022). Competing demands from clinical duties, administrative responsibilities, and training requirements leave minimal time for research activities (Jabonete and Roxas 2022, Sanjari et al. 2015). Additionally, the lack of a structured research‐practice career framework and limited organisational support further restricts engagement (Jabonete and Roxas 2022; Berthelsen and Hølge‐Hazelton, 2021). Other challenges include limited access to research resources, inadequate training in research methodologies, and resistance to change within clinical environments (Berthelsen and Hølge‐Hazelton 2021). These obstacles contribute to low confidence among nurses in undertaking research, thereby hindering both professional growth and advancements in nursing practice.

Nurses can participate in research at multiple levels, including leading studies, supporting investigations and implementing findings into practice. Organisational support is a key enabler of research engagement (Boaz et al. 2015). However, many nurses report a lack of encouragement from leadership and peers, which discourages participation (Konwar and Kalita 2018). Investing in research capacity‐building initiatives, such as education, mentorship programmes and interdisciplinary collaborations, can strengthen nurses' research skills and foster a research‐driven culture (Olive et al. 2022). Establishing research infrastructure, including dedicated research teams, access to academic databases and protected time for research, can further support engagement. Leadership also plays a critical role in facilitating research participation, providing resources and ensuring that nursing research is valued within healthcare organisations (Johantgen et al. 2017; Powers 2020).

Although nurses’ research engagement varies across healthcare systems, there is growing recognition of its importance. In the United Kingdom, nursing research has gained increased visibility, particularly regarding evidence implementation and clinical decision‐making. However, nurse‐led research remains less common. To strengthen nursing research, the Chief Nursing Officer (CNO) for England introduced the “Making Research Matter” strategy in 2021 (Chief Nursing Officer 2021). This strategic plan outlines a vision for embedding nursing research into everyday practice, ensuring that nurses lead, contribute to and apply research to improve patient care. The strategy is built on five key themes, and these are: (i) aligning nurse‐led research with public need (person‐centred research); (ii) releasing nurse's research potential; (iii) building the best research system (Building capacity and capability); (iv) developing future nurse leaders of research; (v) digitally enabled nurse‐led research (digital capacity). A core priority of the third theme is to increase the visibility and impact of nurse‐led research across the NHS and social care. A key step in operationalising this strategy is to assess the current level of research readiness across NHS organisations in England.

Organisational readiness is fundamental to successfully implementing nursing research capacity‐building initiatives, particularly in environments focused on evidence‐based practice. Self‐assessment tools play a crucial role in evaluating research readiness, identifying barriers and guiding organisational improvements. While various conceptual frameworks and tools have been proposed for research engagement evaluation (Cooke 2021), no validated tool currently exists in the UK to assess organisational research culture, capacity and capability within nursing. Additionally, no tool specifically aligns with the CNO's research strategy, highlighting a gap in assessing and strengthening research infrastructure within healthcare organisations.

To address this need, the present study, developed in collaboration with the Department of Health, aims to create a comprehensive research readiness assessment tool. This initiative serves as a foundation for future work in enhancing nursing research engagement across healthcare settings. By developing an appropriate and context‐specific tool, healthcare organisations can systematically assess, benchmark and improve their research culture, ultimately fostering a research‐active nursing workforce and advancing patient care outcomes.

### 1.1 Aim of Study

This study presents the findings of a structured Delphi consensus‐building exercise designed to develop a tool for assessing organisational readiness to support nursing engagement in research. The objective was to establish key organisational indicators that facilitate research capacity development (RCD) within the nursing workforce. The development process was informed by RCD mechanism theory, existing literature, and experiential knowledge, ensuring that the indicators are evidence‐based, contextually relevant and aligned with the needs of nursing research and practice.

## 2 Methods

### 2.1 Research Design

This study adhered to the Standards for Reporting Qualitative Research guidelines (O'Brien et al. 2014). To develop baseline statements, we conducted a comprehensive literature review and engaged in reflective discussions to identify key indicators for organisational readiness. We then employed the Delphi method, an iterative and structured consensus‐building approach, to refine these statements and achieve expert agreement (Diamond et al. 2014). The Delphi technique has been widely applied in healthcare service design and provision, providing a systematic method for gathering expert opinions on complex or emerging topics (Salway et al. 2019). The Delphi process typically begins with the development of open‐ended statements or a loosely structured questionnaire, followed by multiple rounds of controlled feedback shared with a predefined panel of participants. Through this iterative approach, participants provide independent responses, and each round refines the statements based on aggregated feedback. The final outcome is a summary report detailing the level of consensus reached on the developed statements and the key dimensions of the issue under consideration.

The Delphi approach has some advantages over other methods of consensus generation, such as focus group discussions (Avella 2016). It ensures anonymity and allows participants to respond individually without being influenced by dominant voices, thereby reducing social desirability bias (Avella 2016; Nasa, Jain, and Juneja 2021). Additionally, it is a cost‐effective and efficient technique, particularly useful for developing consensus in emerging or contentious areas of healthcare research where limited evidence exists (Shang 2023). Advances in digital collaboration platforms and real‐time data analysis software have further enhanced the Delphi process, allowing experts to contribute remotely across different locations (McPherson et al. 2018). These technological innovations have improved data processing speed, increased participant engagement and reduced dropout rates, ultimately strengthening the reliability and robustness of consensus generation in this study.

### 2.2 Ethical Considerations

Ethical approval for this study was obtained from the University of Sheffield Research Ethics Committee. All participants received a detailed information sheet via email and within the online survey platform, outlining the study's purpose, procedures and their rights as participants. Before participation, individuals were given the opportunity to ask questions and seek clarification. Informed consent was obtained through the online system, requiring participants to confirm their understanding of the study before proceeding. Additionally, for those involved in the consensus‐building workshops, further written confirmation of consent was collected at the start of each session to ensure ongoing voluntary participation.

#### 2.2.1 The Expert Working Group (EWG)

The research team was supported by a working group of nine experts from nursing, Allied Health Professions and research management. Five members were part of a Community of Practice with expertise in using organisational strategy to support RCD in nursing and adapting organisational‐level indicators (Sarre and Cooke 2009) as part of a National Institute for Health and Care Research (NIHR) collaboration. Some group members also had national roles in education, digital innovation, and R&D delivery. Three members were part of the NIHR 70@70 initiative (National Institute for Health and Care Research, 2019), which aimed to strengthen the research voice and influence of nurses and midwives in health and social care settings.

This EWG played a key role in identifying and recruiting Delphi panel participants, ensuring a diverse and informed panel. They provided guidance on questionnaire development, consensus criteria and the decision to conduct a third Delphi round. Additionally, the group contributed to the final report submitted to funders, offering critical feedback and strategic direction throughout the study. Their involvement ensured the rigour, relevance and applicability of the research findings, acting as a sounding board for the research team at every stage of the project.

## 3 The Delphi Panel Sample

The Delphi panel participants were identified through collaboration with the EWG using purposive sampling. Email invitations were sent via both targeted and generic email lists, providing details of the study, researcher contact information, an information sheet and a consent form. Participants were invited to contribute to all three online rounds and the final consensus conference. The final panel represented diverse professional backgrounds including nurses, managers and research leaders from various clinical settings across England.

Table 1 provides a detailed breakdown of participant demographics, ensuring transparency in the representation of different healthcare sectors and expertise areas. This broad and multidisciplinary participation strengthened the validity and applicability of the consensus findings.

**TABLE 1:** Socio‐demographic characteristics of participants.

	Participants who performed ranking in rounds 1–3 (*N* = 37)
Age	
Under 20	
20–29	
30–39	04
40–49	13
50–59	17
60+	3
Gender	
Male	4
Female	29
Working in current role	
Less than 1 year	3
1–5 years	12
6–10 years	6
Over 10 years	16
Role	
Nurse	26
Midwife	3
Allied health professional	2
Academic	2
Research manager	2
Health visitor	1
Part of the healthcare system you currently work	
Primary/community	11
Secondary	22
University/ARC National Strategy role	2
Charity	2
Qualification	
Degree (BSc and BA)	3
Postgraduate	16
PhD/Doctorate	18

### 3.1 Developing a Delphi Questionnaire

Prior to undertaking the study, draft organisational indicators to support RCD in the nursing workforce were shaped by the theory of RCD mechanisms, the existing literature in this field, experiential knowledge and relevance for nursing (Cooke 2021; Cooke, Gardois, and Booth 2018). These indicators were further refined through collaboration with ACORN (Addressing Capacity in Organisations to do Research Network), a regional Community of Practice that had previously adapted organisational RCD indicators to align with NHS research strategies (Sarre and Cooke 2009). To ensure completeness, relevance and alignment with the CNO's research strategy, the EWG reviewed and refined the draft SORT.

The Delphi questionnaire consisted of five sections with 85 statements (another four statements were added in Round 2, making a total of 89 statements), as outlined in Table 2. Each section corresponded to a key pillar of the national strategic research plan for England, called ‘Making Research Matter (2021)’. Participants evaluated the inclusion of each statement in the final tool using a five‐point Likert scale (strongly disagree, disagree, unsure, agree and strongly agree). Additionally, after each subsection, participants were given the opportunity to suggest wording modifications and provide open‐ended feedback on any aspects they wished to bring to the research team's attention.

**TABLE 2:** Statements that reached consensus in Delphi rounds.

		Round 1	Round 2	Round 3
**A1. Infrastructure Statements**				
1	Our organisation has an up‐to‐date list of national, regional and organisational research priorities developed with nurses and service users/carers. These are reviewed every three years.	‐	‐	‐
2	Our research priorities are used to determine nurses’ involvement in leading, delivering, supporting and participating in research.	‐	‐	‐
3	Our organisation has active research/knowledge exchange partnerships with academics where nurses can co‐produce research ideas together with academics. These partnerships make successful applications for funding.	82.1		
4	Our organisation works with others in the health and care system to undertake service development and innovation along care pathways.		76.2	
5	Our organisation works closely with academic departments in universities (by developing honorary contracts, joint posts and collaborations) to develop research questions.		95.2	
6	We have joint posts with academic departments in universities whose aim is to develop research questions based on local population needs and priorities, and undertake research in collaboration with practitioners.	71.4		
7	We have productive (research active) links with parts of the NIHR infrastructure (e.g., Clinical Research Network (CRN), NIHR clinical research facilities, ARC, BRC and Schools for Primary care, public health and social care)	85.7		
8	We have access to and are actively engaged with PPIE networks that are both internal and external to our organisations in order to support the full spectrum of research.	89.3		
9	We have nurses with skills to support PPIE networks, both internal and external to our organisation.	78.6		
**A2. Delivering people‐centred portfolio research as part of clinical nursing practice**				
10	Clinical nurses within our organisation (within the research department and wider clinical settings) use their expertise to deliver research, including portfolio (commercial and non‐commercial) research.	78.6		
11	Nurses who use their expertise to deliver research within a wider interdisciplinarity team have their contribution recognised (e.g. through acknowledgement, co‐authorship in papers and outputs)	71.4		
12	We have a cohort of clinically based nurses who are confident and skilled to contribute to the delivery of portfolio research based on priorities, and we record and review this activity.		76.2	
13	We support nurses working at an enhanced advanced and consultant level of practice to develop their research delivery skills in order to act as PIs on commercial and non‐commercial portfolio studies.	78.6		
**A3. Releasing resources to develop and undertake people‐centred research**				
14	Our organisation has access to ‘seed’ funding (internal or external to the organisation), which is available and used by nurses to undertake pilot and preliminary work to support applications for research funding.	85.7		
15	Protected time is available for clinical nurses to support research and innovation.	75.0		
16	There are resources (e.g. time and/or funds) to support PPIE involvement to identify and develop research.	85.7		
17	We develop impact stories from projects where nurses support, participate or lead research and where such projects have made a difference to services or service users.	78.6		
18	We collect case studies of where PPIE involvement has made a difference to research in our organisation.	75.0		
19	We actively communicate to the nursing workforce, clinical managers and executive team about how the involvement of nurses in research has made a difference to services and people.	85.7		
20	We encourage nurses to take part in research leadership and advisory activities outside our organisation (e.g. sitting on ethics committees, funding committees, editorial boards and reviewing papers).		71.4	
21	We encourage nurses who take part in research leadership and advisory activities outside our organisations to share the learning from these activities within the organisation.		85.7	
22	Nurses within our organisation work with professional organisations and national and regional policy structures to influence the research agenda.	82.1		
**B1. Identifying potential**				
23	Research is included as part of the nursing workforce's annual appraisal process. This is used to identify talent and nurture research career development linked to an individual's stated ambition.			
24	Managers are given training and resources to help direct their staff during appraisal, including advice on research training opportunities, research delivery, research use and service improvements.			
25	Managers know where to direct nursing staff to get research development support, including how to access a range of clinical/practitioner research pathways.	71.4		
**B2. Skills‐based training**				
26	Our Information/library services provide information to nurses, including training, networking, social media platforms and research funding opportunities.		76.2	
27	Nurses have access to, and use, library services, which provide training on searching and appraising the literature.	92.9		
28	Our training strategy includes training, supporting and participating in research and research skills.	78.6		
29	Our training strategy is shaped by a training needs analysis informed by the appraisal system.			
30	Nurses have access to and utilise research learning opportunities within our organisation delivered through our R&D department, service development, education or training departments.	85.7		
31	There are active and regular uni‐ and multiple professional research interest fora (e.g. journal clubs, communities of practice and research interest groups) run within our organisation attended by nurses.	78.6		
32	We have a system to ‘talent spot’ and support individuals who are active in service development/QI to process on to research.	75.0		
**B3. Facilitating environment**				
33	Nurses are members of research‐relevant communities of practice, research networks, shared professional decision‐making and interest groups within and outside our organisation.	75.0		
34	Nurses with our organisation use and work with other parts NIHR infrastructure (RDS, CRN and ARC).	75.0		
35	We have research role models and named research leaders within our organisation.	92.9		
36	There are methods of identifying and celebrating success in research, service transformation based on success and in adopting and translating research findings.	82.1		
37	Our nurses have access to experts who can advise on developing project proposals.		100	
38	We provide ‘research advice sessions’ where nurses can explore ideas for project development.	85.7		
39	We provide peer writing groups.			
40	We provide support to help nurses navigate the funding submissions, ethics and governance systems.	92.9		
41	Our finance departments can cost research project involvement appropriate for external funding applications.	92.9		
**B4. Experiential learning**				
42	Nurses have access to and use research‐related shadowing and placement opportunities.	75.0		
43	We have an active research‐related mentorship programme.	75.0		
44	Nurses in our organisation have access to and use research secondments.		71.4	
45	Our organisation is part of research collaborations that offer research roles to nurses.	71.4		
46	There are mechanisms to identify internship and fellowship opportunities, and we mentor nurses to successfully apply for these.	85.7		
**B5. Releasing resources**				
47	Nurses who have research in their job description have time specifically allocated in their work plan to do this.			
48	We have infrastructures and governance procedures to appropriately utilise awarded grant funding in the manner intended (e.g. protected time and spending decisions).	89.3		
49	Nurses can access resources to attend and present at internal and external conferences and seminars.	89.3		
**C1. Strategy and processes**				
50	Our organisation has a mission statement that includes an ambition to do research as a core activities	82.1		
51	Our organisation has a strategic document to support research capacity development for nursing. This document has a related delivery plan.	89.3		
52	The research capacity delivery plan aims to maximise the use, delivery, collaboration and co‐production and nurse leadership in research.	71.4		
53	The research capacity delivery plan is operationalised across different departments, wider inter‐professional teams and relevant wider health system partners (e.g. ICS).			
**C2. Recognition and reward**				
54	Our organisation runs a series of research events, seminar series and conferences where nurses can share their achievements and gain experience in presenting.	89.3		
55	We have a dedicated database of projects that are nurse‐led or where nurses have made a contribution.		76.2	
56	We have a dedicated database of nurse‐led publications (e.g, articles, book chapters, blogs).			
57	We offer recognition awards to nurses for their research achievements.		71.4	
58	We provide good news stories of research in our internal and external communications.	92.9		
**C3. Monitoring and planning**				
59	There is a process to monitor ambition and training support expressed through our annual appraisal process.			
60	There is a process to monitor ambition and training support expressed through our annual appraisal process we use this to shape the support provided.			
61	We monitor research supervision and successful project development and delivery.	75.0		
62	We monitor our academic partnerships and links with academia to identify their impact.			
63	We audit the research element of job descriptions to monitor actual research activity.			
64	We audit research culture and research support amongst nurses.			
65	We conduct and analyse exit interviews, particularly if their onward destination is in academia.			
66	We audit the research ambition of nurses through appraisal and track progress. Review enablers and blockages.			
**D1. Communication**				
67	Our marketing and workforce recruitment publicity includes our ambition to undertake and deliver research as a core activity.	75.0		
68	Our communications strategy includes research as a career choice in our organisation.			
69	We use our communication channels (internal and external) to share nurse‐led research case study examples, talking heads about nurse research involvement, and impact examples to illustrate research career pathways for nurses.	82.1		
**D2. Research as core business**				
70	Research and development opportunities are included in our induction process.	85.7		
71	We offer pre‐registration and continuing professional development research placement opportunities.	82.1		
72	We review how the research pillar of practice is operationalised.		71.6	
73	We have clear and well‐articulated research career pathway options for nurses that can be used in annual appraisals.			
74	We have clear and well‐articulated research career pathway options for nurses that can be captured in the 3‐yearly NMC revalidation.			
75	We provide opportunities to use research skills at post‐master's (ACP roles) and post‐doctoral levels (nurse consultant roles).	71.4		
**E1. Digital‐enabled research**				
76	We provide training to nurses to help them develop knowledge and skills to understand digital technologies to enable them to practise effectively in a digitally enabled environment.			75
77	We provide training to nurses to help them develop the knowledge and skills they need to use and interpret data that will enable them to make data‐driven improvements to care (using audit, service evaluation or research).			87.5
78	We have digital nurse leaders in place who can provide advice and guidance in the use of digital technology in‐service development and research.			75
79	We have the infrastructure to support digital data collection to inform need, evaluate services and undertake research in compliance with legal and ethical requirements and patient confidentiality.	78.6		
80	We have the infrastructure to support visualisation of data using business intelligence tools.			75
81	We have relevant information governance arrangements, including data‐sharing agreements in place that support data sharing with health care system partners and academic partners that support nurse‐led research.	85.7		
82	We have the internal structure that facilitates, supports and enables nurse‐led digital innovation.			87.5
83	We have effective partnerships with technology suppliers to support digital developments and innovation that meet the needs of nurses and are fit for purpose.			87.5
84	We collect and share case study examples of improvements to care and research using technology.			75
**F1: New statements**				
85	In our organisation, nursing research capacity development is supported and advocated at the executive board level.		71.4	
86	In our organisation, there is a nurse at the executive board level who advocates for research.			
87	Senior nurses within our organisation support and encourage nurses, at all levels, to get involved in research.		76.2	
88	Our research and development department includes nurse leaders who can influence the department's strategic direction and priorities.		85.7	
89	Our workforce and training plans identify clinical research opportunities, and there is a process and mechanism in place to support nurses who want to do this.			

### 3.2 Data Collection and Analysis

The Delphi exercise was conducted between March and June 2022 and consisted of three survey rounds (administered via Google Forms) and four online consensus workshops with the EWG using a semi‐structured discussion approach.

*Round 1*: The first Delphi survey was distributed to 43 panel participants, containing 84 statements across five thematic areas, along with an open‐ended section for additional input. To mitigate attrition and response bias, which are common in Delphi studies, participants received two email reminders within the two‐week response period.

*Analysis following Round 1*: Responses were collated and analysed using MS Excel. The weighted mean of responses for each statement (using a five‐point Likert scale where ‘strongly disagree’ was 1 and ‘strongly agree’ was 5) and the percentage of participants who agreed with the statement (‘agree’ or ‘strongly agree’) were calculated. Graphs illustrating the spread of responses were also produced and inspected. Open‐ended feedback from this round was also reviewed, and minor amendments were made to ensure clarity of statements where needed, and five additional statements suggested by participants were included in round two. At the end of Round 1, 47 statements met the ≥70% consensus threshold and were retained for the final tool. The remaining 37 statements, along with the five new statements, were advanced to Round 2.

*Round 2*: Prior to Round 2, all participants from Round 1 received individualised feedback, including summary tables listing for each statement (i) the weighted average, (ii) the percentage of all participants who disagreed, (iii) the percentage of all participants who agreed and (iv) the percentage of all participants who were unsure.

Participants were then asked to re‐rank 42 statements (including 27 from previous rounds and five new statements) using the same five‐point Likert scale. The survey remained open for three weeks to allow sufficient time for responses.

*Round 3*: An additional Delphi round was conducted to focus on digitally enabled research, a section where consensus was notably lower. After Round 2, only two out of nine statements in this section achieved the ≥70% consensus threshold, with a high proportion of “unsure” responses (see Table 2).

Following concerns raised by the EWG, it was concluded that the limited expertise in digital research within the original Delphi panel may have contributed to the uncertainty. To address this, the EWG recommended engaging a specialised panel with expertise in digitally enabled research. Using the same Delphi process, a third survey was distributed to 15 identified digital research experts, of whom eight completed this round. This additional step ensured greater validity and relevance in the Digitally Enabled Research section of the final tool.

## 4 Findings

### 4.1 Participants

As mentioned earlier, a total of 43 participants meeting the inclusion criteria were initially invited to join the Delphi panel, all of whom agreed to participate. Of these, 28 completed Round 1, yielding a 65% response rate. Round 2 had 21 participants, including one new participant, achieving a 75% response rate. For Round 3, which specifically focused on the Digitally Enabled Research section, 15 additional participants with expertise in digital capacity were invited, of whom eight responded, resulting in a 53.3% response rate. In total, 58 individuals were invited to participate in the Delphi process, with 37 contributing across all three rounds.

Additionally, nine participants engaged in four online consensus‐building workshops, offering expert insights and strategic guidance. The Delphi process resulted in consensus on 67 statements, forming the foundation of the SORT prototype, as presented in Table 3. The following sections detail the thematic findings, highlighting key outcomes and areas requiring further refinement.

**TABLE 3:** Selection of statements in the final SORT.

Section of the questionnaire linked to the CNO objectives	Number of statements reached consensus After round 1	Number of statements reached consensus After round 2	Number of statements reaching consensus After round 3	Total number of statements to be included in SORT
A. Developing people‐centred research	15/22	4/10		19/22
B. Releasing potential	19/27	3/ 8		22/27
C. Research system	6/17	2/11		8/17
D. Careers (developing leaders)	5/9	1/6		6/9
E. Digital enabled research	2/9	0/7	7/7	9/9
F. New statements		3/5		3/5
**Total included in each round**	**47**	**13**	**7**	**67/89**

Five new statements were added in round 2, and out of these, 3 were added in the final tool in section A.

### 4.2 People‐Centred Research

This theme emerged as the largest section, with 22 statements achieving consensus. These statements highlight the importance of collaboration across the health and care system, emphasising partnerships with academic institutions and the integration of Public and Patient Involvement and Engagement (PPIE) into research processes. Leadership support was a recurring focus, with statements underscoring the need for organisational commitment to champion research initiatives. This theme aligns with the principles of patient‐centred care, ensuring that research activities are practical, relevant and integrated system‐wide.

### 4.3 Releasing Potential

Twenty‐two statements reached a consensus on this theme, underscoring the critical role of skills development, experiential learning and structured facilitation in building research capacity. Statements emphasised the need for mentorship programmes, tailored training and accessible resources to support nurses in engaging with research. Additionally, this theme highlighted the importance of recognising and rewarding research contributions, ensuring they are aligned with organisational priorities to encourage sustained participation.

### 4.4 Research Systems

This theme focused on organisational strategies for R&D, recognition mechanisms for research contributions, and systems for monitoring progress. Eight statements achieved consensus, but key areas such as progress tracking and evaluating research impact did not meet the threshold. These findings suggest the need for further refinement to establish effective monitoring practices for research readiness at an organisational level.

### 4.5 Careers

The Careers section contained six statements that achieved consensus. These statements emphasised embedding research as a core organisational function through mechanisms such as continual professional development, induction programmes and structured career pathways. Notably, statements on using annual appraisals to identify research potential (52% agreement) and providing training for managers to support research careers (57% agreement) did not reach a consensus. These results suggest that additional focus is needed to integrate research into routine workforce management processes.

### 4.6 Digitally Enabled Research

This theme presented the greatest challenges, with only two out of nine statements initially reaching consensus in the first round. The high proportion of ‘unsure’ responses suggested a lack of confidence or knowledge among the original panel regarding digital health and innovation. To address this, a third Delphi round engaged a national group of digital capacity experts, leading to consensus on seven additional statements. These focused on building digital capacity, leveraging technology for research and integrating digital tools into organisational practices. Despite this progress, digitally enabled research remains an area requiring further development.

Out of the five new statements introduced in Round 2, three achieved consensus and were incorporated into the final tool. The finalised SORT prototype consists of 70 statements (Table 4), covering all five thematic areas of the CNO research strategy. The tool provides a comprehensive assessment framework, addressing key aspects of research engagement, including evidence‐based practice, workforce development, leadership and digital innovation.

**TABLE 4:** SORT tool: prototype.

A.	A. Developing people‐centred research
	**Infrastructure**
A1	Our organisation has active research/ knowledge exchange partnerships with academics where nurses can co‐produce research ideas together with academics. These partnerships make successful applications for funding.
A2	Our organisation works with others in the health and care system to undertake service development and innovation along care pathways.
A3	We have Joint posts with academic departments in universities whose aim is to develop research questions based on local population needs and priorities and undertake research in collaboration with practitioners.
A4	We have productive (research active) links with parts of the NIHR infrastructure (e.g., Clinical Research Network (CRN), NIHR clinical research facilities, Applied Research Collaborations (ARC), Biomedical Research Centres (BRC); Schools for Primary care, public health, and social care).
A5	We have access to and are actively engaged with Public and Patient Involvement & Engagement (PPIE) networks that are both internal and external to our organisations in order to support the full spectrum of research.
A6	We have nurses with skills to support PPIE networks, both internal and external to our organisation.
	**Delivering people‐centred portfolio research as part of clinical nursing practice**
A7	Clinical nurses within our organisation (within the research department and wider clinical settings) use their expertise to deliver research, including portfolio (commercial and **non‐commercial**) research.
A8	Nurses who use their expertise to deliver research within a wider interdisciplinarity team have their contribution recognised (e.g., through acknowledgement, co‐authorship in papers and outputs).
A9	We have a cohort of nurses who are confident and skilled to contribute to the delivery of portfolio research based on priorities, and we record and review this activity.
A10	We support nurses working at an enhanced advanced and consultant level of practice to develop their research delivery skills in order to act as principal investigators (PIs) on commercial and non‐commercial portfolio studies.
	**Releasing resources to develop and undertake people‐centred research**
A11	Our organisation has access to ‘seed’ funding (internal or external to the organisation), which is available and used by nurses to undertake pilot and preliminary work to support applications for research funding.
A12	Protected time is available for clinical nurses to support research and innovation.
A13	There are resources (e.g., time and/or funds) to support PPIE involvement to identify and develop research.
	**Measuring impact to show how research makes a difference**
A14	We develop impact stories developed from projects where nurses support, participate or lead research and where such projects have made a difference to services or service users.
A15	We collect case studies of where PPIE involvement has made a difference to research in our organisation.
A16	We actively communicate to the nursing workforce, clinical managers and executive team about how the involvement of nurses in research has made a difference to services and people.
	**Influence and leadership**
A17	We encourage nurses to take part in research leadership and advisory activities outside our organisation (for example, sitting on ethics committees, funding committees, editorial boards and reviewing papers).
A.18	We encourage nurses who take part in research leadership and advisory activities outside our organisations to share the learning from these activities within the organisation.
A19	Nurses within our organisation work with professional organisations and national and regional policy structures to influence the research agenda.
A20	In our organisation, nursing research capacity development is supported and advocated at the executive board level.
A21	Senior nurses within our organisation support and encourage nurses, at all levels, to get involved in research.
A22	Our research and development department includes nurse leaders who can influence the department's strategic direction and priorities.

	B. Releasing Potential
	**Identifying potential**
B1	Managers know where to direct nursing staff to get research development support, including how to access a range of clinical/practitioner research pathways.
	**Skills‐based training**
B2	Our information/library services provide information to nurses, including training, networking, social media platforms and research funding opportunities.
B3	Nurses have access to, and use, library services, which provide training on searching and appraising the literature.
B4	Our training strategy includes training, supporting and participating in research and research skills.
B5	Nurses have access to and utilise research learning opportunities within our organisation delivered through our R&D department, service development, education or training departments.
B6	There are active and regular university and multiple professional research interest fora (e.g., journal clubs, communities of practice and research interest groups) run within our organisation attended by nurses.
B7	We have a system to ‘talent spot’ and support individuals who are active in‐service development/QI to process on to research.
	**Facilitating environment**
B8	Nurses are members of research‐relevant communities of practice, research networks, shared professional decision‐making and interest groups within and outside our organisation.
B9	Nurses with our organisation use and work with other parts NIHR infrastructure (RDS, CRN, and ARC).
B10	We have research role models and named research leaders within our organisation.
B11	There are methods of identifying and celebrating success in research, service transformation based on success and in adopting and translating research findings.
B12	Our nurses have access to experts who can advise on developing project proposals.
B13	We provide ‘research advice sessions’ where nurses can explore ideas for project development.
B14	We provide support to help nurses navigate the funding submissions, ethics and governance systems.
B15	Our finance departments can cost research project involvement appropriate for external funding applications.
	**Experiential learning**
B16	Nurses have access to and use research‐related shadowing and placement opportunitie**s**.
B17	We have an active research‐related mentorship programme.
B18	Nurses in our organisation have access to and use research secondments.
B19	Our organisation is part of research collaborations that offer research roles to nurses.
B20	There are mechanisms to identify internship and fellowship opportunities, and we mentor nurses to successfully apply for these.
	**Releasing resources**
B21	We have infrastructures and governance procedures to appropriately utilise awarded grant funding in the manner intended (e.g., protected time and spending decisions).
B22	Nurses can access resources to attend and present at internal and external conferences and seminars.

	C. Systems
	**Strategy and processes**
C1	Our organisation has a mission statement that includes an ambition to do research as a core activity.
C2	Our organisation has a strategic document to support research capacity development for nursing. This document has a related delivery plan.
C3	The research capacity delivery plan aims to maximise the use, delivery, collaboration and co‐production and nurse leadership in research.
	**C2. Recognition and reward**
C4	Our organisation runs a series of research events, seminar series and conferences where nurses can share their achievements and gain experience in presenting.
C5	We have a dedicated database of projects that are nurse‐led or where nurses have made a contribution.
C6	We offer recognition awards to nurses for their research achievements.
	**C3. Monitoring and planning**
C7	We monitor research supervision and successful project development and delivery.
C8	We provide good news stories of research in our internal and external communications.

	D. Careers
	**Communication**
D1	Our marketing and workforce recruitment publicity includes our ambition to undertake and deliver research as a core activity.
D2	We use our communication channels (internal and external) to share nurse‐led research case study examples, talking heads about nurse research involvement, and impact examples to illustrate research career pathways for nurses.
	**D2. Research as core business**
D3	Research and development opportunities are included in our induction process.
D4	We offer pre‐registration and continuing professional development research placement opportunities.
D5	We review how the research pillar of practice is operationalised.
D6	Our career pathways provide opportunities to use research skills and experience of leadership at post‐master's (ACP roles) and post‐doctoral levels (nurse consultant roles).

	E. Digital‐enabled research
	**E1. Digital‐enabled research**
E1	We have the infrastructure to support digital data collection to inform needs, evaluate services and undertake research in compliance with legal and ethical requirements and patient confidentiality.
E2	We have relevant information governance arrangements, including data sharing agreements, in place that support data sharing with health care system partners and academic partners that support nurse‐led research.
E3	We provide training to nurses to help them develop knowledge and skills to understand digital technologies to enable them to practise effectively in a digitally enabled environment.
E4	We provide training to nurses to help them develop the knowledge and skills they need to use and interpret data that will enable them to make data‐driven improvements to care (using audit, service evaluation or research)
E5	We have digital nurse leaders in place who can provide advice and guidance in the use of digital technology in‐service development and research.
E6	We have the infrastructure to support the visualisation of data using business intelligence tools.
E7	We have the internal structure that facilitates, supports and enables nurse‐led digital innovation.
E8	We have effective partnerships with technology suppliers to support digital developments and innovation that meet the needs of nurses and are fit for purpose.
E9	We collect and share case study examples of improvements to care and research using technology.

## 5 Discussion

This study developed the SORT tool through a structured Delphi process and expert consultation, achieving consensus on 67 statements across five thematic areas aligned with the CNO's research strategy. The iterative Delphi approach, supplemented by targeted expert workshops, ensured that SORT reflects both evidence‐based priorities and practical insights from stakeholders.

The strongest consensus was achieved in the people‐centred research and releasing potential domain, reinforcing the idea that institutional support, skills development and collaborative opportunities significantly influence research engagement (Janerka et al. 2024; Olive et al. 2022). These findings highlight the importance of leadership investment in research training and organisational infrastructure to cultivate a strong research culture within healthcare settings.

Both themes are highly relevant for the practice context within the health system, potentially stimulating and harnessing motivation and relevance for practice as well, to enable this. The ‘People‐centred research’ items include developing outward engagement with others in the health and care system, as well as supporting links with academia and PPIE. These include many items linked to leadership within the organisation and wider contexts. Prior studies have also demonstrated that partnerships with universities and research organisations not only enhance research capacity but also provide structured mentorship opportunities that encourage sustained engagement in research activities (Boaz et al. 2015).

Additionally, SORT integrates evidence‐based practice (EBP) as a pathway into research engagement, encompassing research utilisation, delivery of commercial and non‐commercial studies and research leadership. By positioning EBP as an entry point, SORT addresses the long‐standing ‘research‐practice gap’ in nursing (Boaz et al. 2015; Tuppal et al. 2019; Berthelsen and Hølge‐Hazelton 2021). This scaffolded approach allows organisations to transition from research use to active research generation, a progression widely recognised in nursing research frameworks (Tuppal et al. 2019).

The ‘Research Systems’ section covers items linked to organisational R&D strategy and planning, recognition and reward and monitoring progress. However, several items around monitoring did not achieve consensus, and this may need further consideration as SORT is used in practice. This aligns with broader concerns in the field regarding the difficulty of measuring research outcomes and translating findings into tangible organisational change (Rees and Bracewell 2019). While previous research suggests that establishing clear performance metrics for research engagement can drive improvement (Cooke 2021), this study stresses the need for more refined strategies to monitor research impact at an organisational level.

The ‘Careers’ section has only six items, but four of these emphasise research practice as a ‘core business’ through mechanisms such as continual professional development, the induction process and career pathways. The item on the use of annual appraisal did not achieve consensus (52% agreement in the second round), and training for managers to support research career advice (57% agreement in the second round). The limited agreement on integrating research into career pathways (e.g., annual appraisals) signals systemic barriers, such as competing clinical priorities and insufficient managerial training, well‐documented in nursing research engagement studies (Jabonete and Roxas 2022; Konwar and Kalita 2018).

One of the more surprising findings was the limited consensus around digitally enabled research, despite its growing importance in modern healthcare settings. The necessity for a third Delphi round specifically for this domain underscores the evolving and complex nature of digital health capabilities within nursing research. Similar difficulties have been reported in prior studies, where digital literacy and infrastructure disparities impact the feasibility and perceived value of digital research tools (Astier et al. 2020). The additional engagement with digital experts in the final round of this study not only improved consensus but also revealed the need for further development in this area.

The EWG provided considerable guidance to co‐produce SORT through providing their expert advice throughout the project, and it is hoped that, through working co‐productively with these experts, we have produced a tool that is more likely to be useful and used based on their advice.

### 5.1 Strengths and Weaknesses of the Study

One of the key strengths of this study is its novelty. To our knowledge, this is the first study to develop a prototype tool to enable the assessment of organisational research capacity‐building ability among nurses, thereby filling a significant knowledge gap. By adhering to best practices for conducting a Delphi exercise, we successfully generated a comprehensive set of statements, achieving satisfactory response and retention rates. A particularly valuable aspect of our approach was the inclusion of a deliberative consensus conference, which allowed for detailed examination of statements lacking agreement and facilitated discussions on appropriate refinements. This process strengthened confidence in the final set of indicators, ensuring they provide a robust foundation for future application and refinement.

While the Delphi method is widely recognised for its structured and iterative approach to expert consensus, it presents challenges in defining and recruiting a sufficiently diverse expert panel and maintaining engagement throughout multiple rounds (Keeney et al. 2006; Keeley et al. 2016). We used purposive sampling, with explicit inclusion criteria developed in collaboration with the project commissioners. However, participant retention was impacted by timing constraints, as data collection coincided with two UK bank holidays and school holiday periods, particularly during Round 2.

The demographic profile of the Delphi panel highlighted an existing trend in nursing research engagement, where research leadership is often linked to seniority and career stage. This reinforces previous findings that nurses and midwives tend to engage in research later in their professional careers, often due to delayed exposure to formal research training and opportunities. Additionally, while the Delphi rounds were largely successful, Digitally Enabled Research emerged as a challenging domain, with statements on digital enablement and monitoring eliciting a higher proportion of ‘unsure’ responses. This suggests a potential expertise gap, which may be attributed to variability in digital literacy and differing levels of institutional digital infrastructure. Similar trends have been observed in other studies, where emerging healthcare technologies often reveal gaps in participant familiarity and confidence (Shang 2023). Future studies should explore targeted strategies for strengthening digital research competencies within nursing and healthcare professions.

Another limitation is that this study was conducted exclusively in England, with this paper reporting only the Delphi phases of the broader research programme. Several methodological factors may have influenced the findings: dropout rates between Delphi rounds, time constraints during data collection, and potential response bias could have affected consensus patterns and outcomes. While Delphi consensus establishes face validity, the instrument requires a comprehensive psychometric evaluation through statistical testing (e.g., factor analysis and reliability testing) to confirm its structural integrity and internal consistency. Although such psychometric testing has been completed and demonstrates satisfactory properties, these results form the basis of a separate publication and are not reported here. The single‐country design and focus on English healthcare contexts may limit generalisability to other healthcare systems, organisational structures or cultural settings. These methodological considerations highlight the importance of rigorous validation studies when adapting SORT for use in different geographical regions, healthcare systems or professional contexts beyond nursing.

#### 5.1.1 Future Research

Future work will involve piloting the tool across NHS organisations in the UK, including devolved nations such as Scotland, Wales and Northern Ireland. This will include a sample of nurses and managers using an online survey version of the SORT tool. Psychometric testing, including exploratory and confirmatory factor analysis, will be conducted to validate the structure and reliability of the tool. Further international adaptation will require cultural tailoring and testing in different health system contexts.

## 6 Conclusion and Recommendations

The development of SORT marks a significant advancement in supporting organisational readiness for nursing research. Aligned with the CNO strategy, it provides a practical framework for embedding research into nursing practice. The Delphi process has established good face validity for the tool, demonstrating its potential to address key areas of RCD within healthcare organisations. However, this represents the first step in the process, and further work is required to explore the tool's reliability and undertake rigorous psychometric testing, including factor analysis. These next steps will be crucial to refine SORT and ensure its robustness for widespread implementation.

## 7 Implications for Nursing Practice and Policy

SORT is designed to be used by nursing and R&D leaders, healthcare executives, managers and policymakers to evaluate and improve organisational readiness. It can inform strategic planning, workforce development and performance benchmarking within healthcare institutions.

The development of the SORT has far‐reaching implications for nursing practice and health policy. For nursing practice, SORT provides a structured framework to integrate research activities into everyday clinical care. By embedding EBP into routine nursing activities, the tool promotes a culture where clinical decision‐making is informed by high‐quality research, ultimately leading to improved patient outcomes and safety. It also supports workforce development by identifying organisational gaps in research capacity, enabling the implementation of professional development programmes, mentorship opportunities and career pathways that empower nurses to engage in and lead research initiatives.

SORT also emphasises the importance of leadership in fostering a research culture. By encouraging nurses to take on leadership roles, it helps drive organisational change and supports collaboration with allied health professionals, academic researchers and PPIE stakeholders. This interdisciplinary approach enhances the quality of research and ensures its relevance to practice. Additionally, the tool addresses the growing importance of digital innovation in healthcare, highlighting the need to build digital literacy and capacity among nurses. This prepares the workforce to effectively leverage technology for research and innovation, ensuring they remain equipped to meet the demands of modern healthcare delivery.

From a policy perspective, SORT offers a valuable mechanism for benchmarking and strategic planning. Policymakers can use it to assess research readiness across healthcare organisations and align these assessments with national goals for nursing research capacity building. The tool translates key objectives of the CNO strategy, Making Research Matter, into actionable organisational indicators, facilitating the operationalisation of person‐centred care, research capacity building and digital innovation strategies.

Furthermore, SORT identifies specific gaps in research infrastructure, such as the need for enhanced funding, leadership training and digital capabilities. This enables policymakers to direct resources to underdeveloped areas, fostering a stronger and more sustainable research environment. By generating high‐quality, practice‐relevant evidence, SORT supports evidence‐based policymaking, ensuring that healthcare policies are informed by robust research. Its focus on PPIE ensures that health policies remain aligned with the needs of patients and service users.

## Author Contributions

Study design: JC, JM and PA. Data collection: JC, JM and PA. Data Analysis: JC, JM and PA. Study supervision: JC. Manuscript writing: All.

## Conflicts of Interest

No conflict of interest.
